# In-hospital percentage BNP reduction is highly predictive for adverse events in patients admitted for acute heart failure: the Italian RED Study

**DOI:** 10.1186/cc9067

**Published:** 2010-06-16

**Authors:** Salvatore Di Somma, Laura Magrini, Valerio Pittoni, Rossella Marino, Antonella Mastrantuono, Enrico Ferri, Paola Ballarino, Andrea Semplicini, Giuliano Bertazzoni, Giuseppe Carpinteri, Paolo Mulè, Maria Pazzaglia, Kevin Shah, Alan Maisel, Paul Clopton

**Affiliations:** 1 Emergency Medicine Department, II Medical School University La Sapienza, Sant'Andrea Hospital, via di Grottarossa, 1039, Roma 00189, Italy; 2 Emergency Medicine Department, San Martino Hospital, University of Genova, l.go R. Benzi, 10, Genova 16132, Italy; 3 Clinical and Experimental Medicine Department, University Hospital of Padova, Via Giustiniani, 2, Padova 35128, Italy; 4 Emergency Medicine Department, I Medical School University La Sapienza, Umberto I Hospital, viale del Policlinico, 155, Roma 00161, Italy; 5 Emergency Medicine Department, Vittorio Emanuele Hospital, via S. Sofia, 78, Catania 95123, Italy; 6 Emergency Medicine Department, Sant'Orsola - Malpighi, University Hospital, Via Albertoni, 15, Bologna 40138, Italy; 7 Emergency Medicine Department, Hospital of Ravenna, Viale Randi, 5, Ravenna 48121, Italy; 8 Division of Cardiology, Department of Medicine Veteran's Affairs San Diego Healthcare System, La Jolla Village Drive, 3350, San Diego 92161 La Jolla, CA 92161, USA

## Abstract

**Introduction:**

Our aim was to evaluate the role of B-type natriuretic peptide (BNP) percentage variations at 24 hours and at discharge compared to its value at admission in order to demonstrate its predictive value for outcomes in patients with acute decompensated heart failure (ADHF).

**Methods:**

This was a multicenter Italian (8 centers) observational study (Italian Research Emergency Department: RED). 287 patients with ADHF were studied through physical exams, lab tests, chest X Ray, electrocardiograms (ECGs) and BNP measurements, performed at admission, at 24 hours, and at discharge. Follow up was performed 180 days after hospital discharge. Logistic regression analysis was used to estimate odds ratios (OR) for the various subgroups created. For all comparisons, a *P *value < 0.05 was considered statistically significant.

**Results:**

BNP median (interquartile range (IQR)) value at admission was 822 (412 - 1390) pg\mL; at 24 hours was 593 (270 - 1953) and at discharge was 325 (160 - 725). A BNP reduction of >46% at discharge had an area under curve (AUC) of 0.70 (*P *< 0.001) for predicting future adverse events. There were 78 events through follow up and in 58 of these patients the BNP level at discharge was >300 pg/mL. A BNP reduction of 25.9% after 24 hours had an AUC at ROC curve of 0.64 for predicting adverse events (*P *< 0.001). The odds ratio of the patients whose BNP level at discharge was <300 pg/mL and whose percentage decrease at discharge was <46% compared to the group whose BNP level at discharge was <300 pg/mL and whose percentage decrease at discharge was >46% was 4.775 (95% confidence interval (CI) 1.76 - 12.83, *P *< 0.002). The odds ratio of the patients whose BNP level at discharge was >300 pg/mL and whose percentage decrease at discharge was <46% compared to the group whose BNP level at discharge was <300 pg/mL and whose percentage decrease at discharge was >46% was 9.614 (CI 4.51 - 20.47, *P *< 0.001).

**Conclusions:**

A reduction of BNP >46% at hospital discharge compared to the admission levels coupled with a BNP absolute value < 300 pg/mL seems to be a very powerful negative prognostic value for future cardiovascular outcomes in patients hospitalized with ADHF.

## Introduction

Heart failure (HF) represents an emerging and growing public health problem [1]. In Europe, patients with a diagnosis of chronic HF represent about 14 million people. The prevalence is 2 to 5% in patients over the age of 65 years, with HF complications above 300,000 per year. It is a frequent cause of adult hospitalization, especially in elderly patients [2-4] and carries a high mortality rate [5]. Acute HF is defined as a rapid onset of signs (tachycardia, tachypnoea, pulmonary rales, pleural effusion, raised jugular venous pressure, peripheral edema, hepatomegaly) and symptoms (breathlessness at rest or on exercise, fatigue, tiredness, ankle swelling) of HF, resulting in the need for urgent therapy. It could present as a new HF or worsening HF in the presence of chronic HF [6]. Although patients appear to be effectively treated during their hospital stay, HF patients often experience relapses of acute decompensation and subsequent re-hospitalization [7]. Recent data have shown that precipitating comorbid factors (e.g., respiratory tract infections, arrhythmias, cardiac ischemia) were associated with more severe clinical outcomes independent of other prognostic factors such as kidney failure or hypertension [8]. The best current understanding suggests that in the setting of volume expansion or pressure overload, the resulting wall stress initiates synthesis of pre-pro-brain natriuretic peptide (BNP) in the ventricular myocardium, although some have questioned the correlation between individual changes in blood volumes and natriuretic peptide levels. The release of BNP results in improved myocardial relaxation and serves an important regulatory role in response to acute increases in ventricular volume by opposing the vasoconstriction, sodium retention, and antidiuretic effects of the activated renin-angiotensin-aldosterone system. Natriuretic peptides, both BNP and n-terminal pro-B-type natriuretic peptide (NT-proBNP), together with other biochemical and instrumental tests, can prove useful in assessing diagnosis, severity and prognosis in patients with acute decompensated HF (ADHF) [9-16]. Serial measurements of BNP could be useful not only in guiding the diagnosis of HF, but also in guiding decisions towards therapy and the evaluation of HF stabilization [16-18]. There is a large body of evidence that natriuretic peptides are independent predictors of total mortality, cardiovascular mortality, and HF hospitalizations in both acute and chronic HF [19-23]. In-hospital BNP changes with HF patients appear to be a strong independent predictor of re-hospitalization and mortality [23,24]. Moreover, repeated BNP measurements in patients admitted to the hospital with ADHF could predict outcomes [24,25], even if serial BNP levels are challenged by several factors (individual variability, spontaneous fluctuations) [26,27]. Cheng and colleagues demonstrated that patients with a significant decrease of BNP at discharge compared with admission BNP levels had better outcomes, whereas BNP levels dropped minimally during hospitalization in patients that were re-hospitalized within 30 days [24]. In addition, Cohen-Solal and colleagues demonstrated that patients with a 30% or higher BNP value from baseline, at follow-up had reduced mortality risk compared with those with little or no BNP decrease [28]. It could be also useful to identify patients with ADHF who, after admission to the emergency department (ED), evidence of a reduction in BNP after acute treatment with diuretics or vasodilators. The aim of our study was to evaluate the role of BNP percentage variations at 24 hours and at discharge time compared with its value at admission in the ED in demonstrating its potential predictive value for future cardiovascular events (deaths and/or re-hospitalizations).

## Materials and methods

A total of 287 consecutive patients (Table 1) admitted to the ED for ADHF were enrolled from eight Italian ED centers (Malpighi University Hospital-Bologna; Vittorio Emanuele Hospital-Catania; S. Martino University Hospital-Genova; Policlinico Federico II University Hospital-Napoli; Policlinico of Padova University Hospital-Padova; Ravenna Hospital-Ravenna; S. Andrea Hospital, University La Sapienza-Roma; Policlinico Umberto I, University La Sapienza-Roma) from January 2006 to November 2007. The diagnosis of ADHF was performed on the basis of current guidelines [6]. The majority of patients (n = 243) had a ADHF as decompensation of chronic HF and the remaining patients (n = 44) had an episode of HF of new diagnosis. Two independent cardiologists reviewed the patients and confirmed the diagnosis of ADHF at discharge. Exclusion criteria were: acute coronary syndromes including myocardial infarction, body mass index of 30 Kg/m^2 ^or higher, renal failure maintained on hemodialysis, or dyspnea due to trauma or other causes. The study conformed to the Helsinki declaration and the study protocol was approved by the local ethical committees of all participating hospitals. Written informed consent for the study was obtained from each patient before entering the study. Documentation of the personal medical history was obtained. Each patient underwent physical examination, electrocardiogram, chest x-ray, arterial blood gas analysis, an echocardiographic exam was optional but the majority of patients (n = 249) underwent an echocardiographic exam at admission with, at least, the evaluation of ejection fraction. Blood tests for hemochromocytometric exam, creatinine, urea, electrolytes, and cardiac enzymes were performed. Test results and therapy were reported by the ED in a case report and the ED physicians were asked to rate the severity of HF by New York Heart Association (NYHA) classification. All data were collected in system software by the coordinating center. Each patient was treated with a standard dosage of nitrates, beta-blockers, angiotensin-converting enzyme (ACE) inhibitors and diuretics accordingly to guidelines for ADHF and assessed by physical exam at admission [6]. Therapy was accurately recorded during the course of hospitalization. All patients enrolled had a venous blood sample collected in an EDTA tube to measure BNP levels at admission in the ED and repeated at 24 hours, and at the time of discharge. BNP measurement was tested in Triage^®^-BNP test device (Biosite-Inverness Medical, San Diego, CA, USA), a single-use fluorescence immunoassay 'ready to use' following the manufacturers recommendations for point-of-care testing. The following concomitant clinical and laboratory parameters were considered for discharge criteria: reduction of dyspnoea, respiratory rate below 30 breaths/min, oxygen saturation above 90%, complete clearance of rales at chest examination, and significant reduction of lower limb edema [18]. Fourty patients were moved from EDs to other hospitals, and their BNP discharge samples were lost, so they were excluded from the statistical analysis. The statistical analysis was performed on 247 patients. Patients' follow-up was performed at 30, 90 and 180 days following discharge from ED. In the 247 remaining patients, follow-up was performed by telephone interviews or visits to outpatient clinic and patients or other family components were asked to clarify if the patients had other re-hospitalizations for dyspnoea or edema or deaths for cardiovascular events. On the basis of the BNP absolute value at the moment of discharge, patients were divided into two pre-specified groups of more than 300 pg/ml or less than 300 pg/ml according to literature data [29-31]. The patients were followed to define the odds ratio (OR) to evaluate what incidence of adverse events occurred in the two groups. Numerical values are presented as medians with interquartile ranges (IQR), as appropriate. Categorical values are presented as numbers and percentages. Receiver-operating characteristic (ROC) curves were created to identify the prognostic value of a drop in percentage of BNP level at 24 hours after hospitalization and a drop in percentage of BNP level at discharge. Optimal cut-off points were defined by maximization the product of sensitivity and specificity. Univariate logistic regression analysis was used to estimate ORs for the various subgroups created. Multivariate logistic regression was utilized to test for the significance of the two BNP indicators (discharge and percentage change) simultaneously and to test for the interaction of these two predictors. For all comparisons, a *P *value less than 0.05 was considered statistically significant. All statistics were calculated with Statistical Package for Social Sciences version 12.0 for Windows (SPSS Inc., Chicago, IL, USA).

**Table 1:** Patient characteristics

Number of patients	247
Age in years (mean ± SD)	76 ± 12
Sex	
Male	118
Female	129
NYHA functional classification	
III	33%
IV	67%
Heart rate beats/minute (mean ± SD)	91 ± 22
Systolic blood pressure mmHg (mean ± SD)	143 ± 29
Diastolic blood pressure mmHg (mean ± SD)	79 ± 16
Respiratory rate breaths/minute (mean ± SD)	25 ± 7
Oxygen saturation %(mean ± SD)	91 ± 6
Ejection fraction %(mean ± SD)	45 ± 13
<50%	59%
>50%	41%
Past medical history %	
Hypertension	70%
Coronary heart disease	45%
Diabetes mellitus	29%
Atrial fibrillation	44%

NYHA, New York Heart Association; SD, standard deviation.

## Results

The characteristics of studied patients are presented in Table 1. The following drugs were administered during hospitalization: intravenous loop diuretics to all patients (furosemide); beta-blockers to 22.4%, ACE inhibitors to 51.0%; angiotensin II receptor blockers, (accordingly to ADHF guidelines) in all centres [6], and digoxin and spironolactone in selected cases; and oxygen delivery to maintain oxygen saturation in more than 90% or patients. Continuous positive air-way pressure was utilized for 12% of the patients. The sample was quite homogeneous across the eight centers. In fact the number of patients in NYHA class III and IV was similar at ED admission. Moreover, also the BNP levels at admission in the eight centers were comparable. The treatment in all centers was quite similar. In fact, comparing BNP percentage decreases at 24 hours and discharge, there was no significant difference among centers. In all study subjects (247 patients) BNP median (IQR) value at admission was 822 (412 to 1390) pg/mL, at 24 hours it was 593 (270 to 1953) pg/mL and at discharge it was 325 (160 to 725) pg/mL. During follow up, there were 78 patients with events among the 247 patients enrolled in the study: seven deaths (one due to a noncardiac cause and six deaths due to ADHF) and 71 hospitalizations for dyspnoea and/or relapse congestion between discharge and 180 days. Table 2 shows the distribution of cardiovascular events at various times of follow-up. The OR for those with a discharge BNP of 300 pg/ml or higher as compared with those below this value was 3.17 (95% CI 1.79-5.60, p < 0.001).

**Table 2:** Events and timing

Time	30 days	90 days	180 days
Re-hospitalizations	28	20	23
Deaths	1	5	1

NYHA, New York Heart Association; SD, standard deviation.

**Figure 1:**
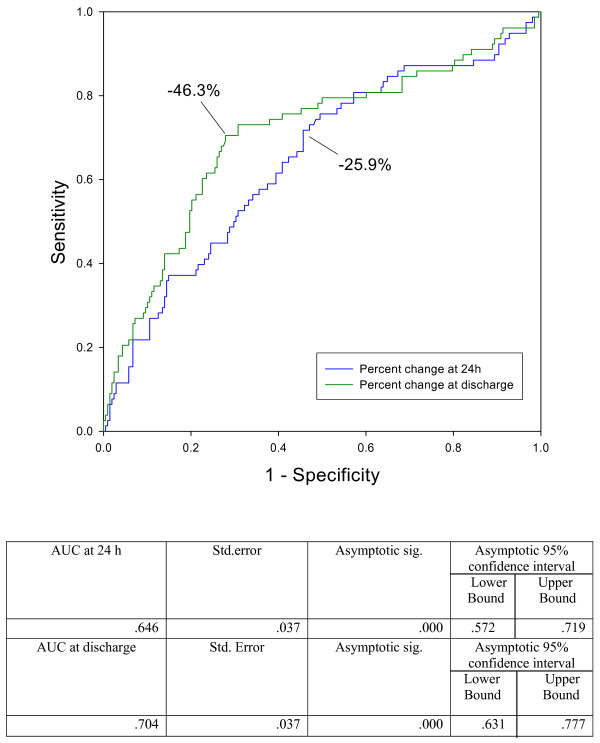
**Receiver operator characteristic curve for percentage change at 24 hours and discharge**. Percentage changes of brain natriuretic peptide at discharge have a higher area under the curve (AUC) than percentage changes at 24 hours, for predicting adverse events.

**Figure 2:**
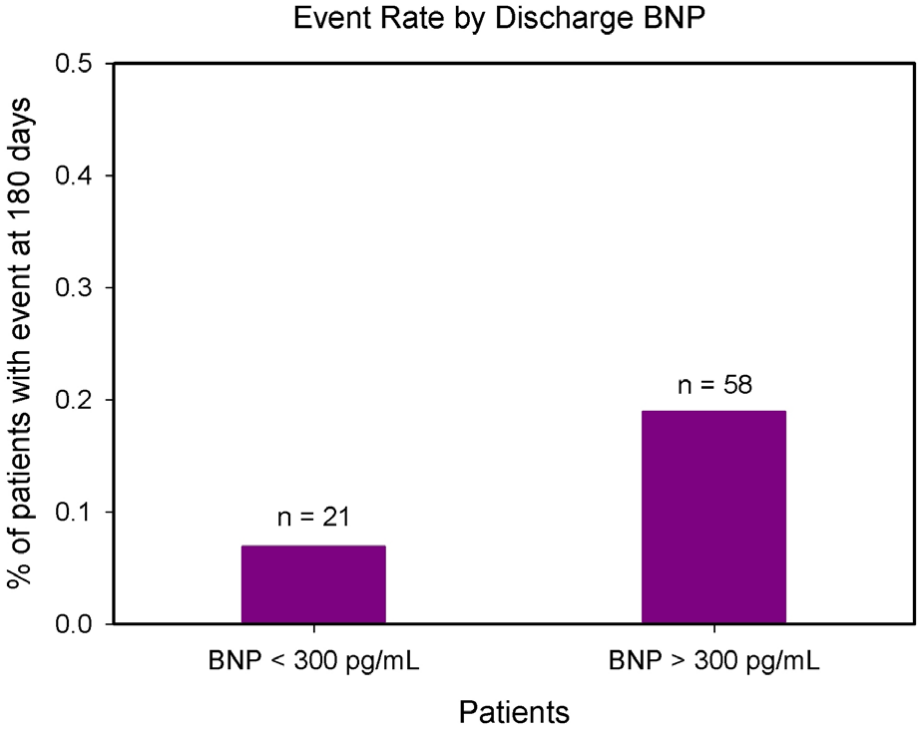
**Event rate by discharge BNP**. Patients with discharge brain natriuretic peptide (BNP) levels above 300 pg/mL had a higher proportion of individuals with adverse events, as compared with patients with discharge BNP value of 300 pg/mL.

**Figure 3:**
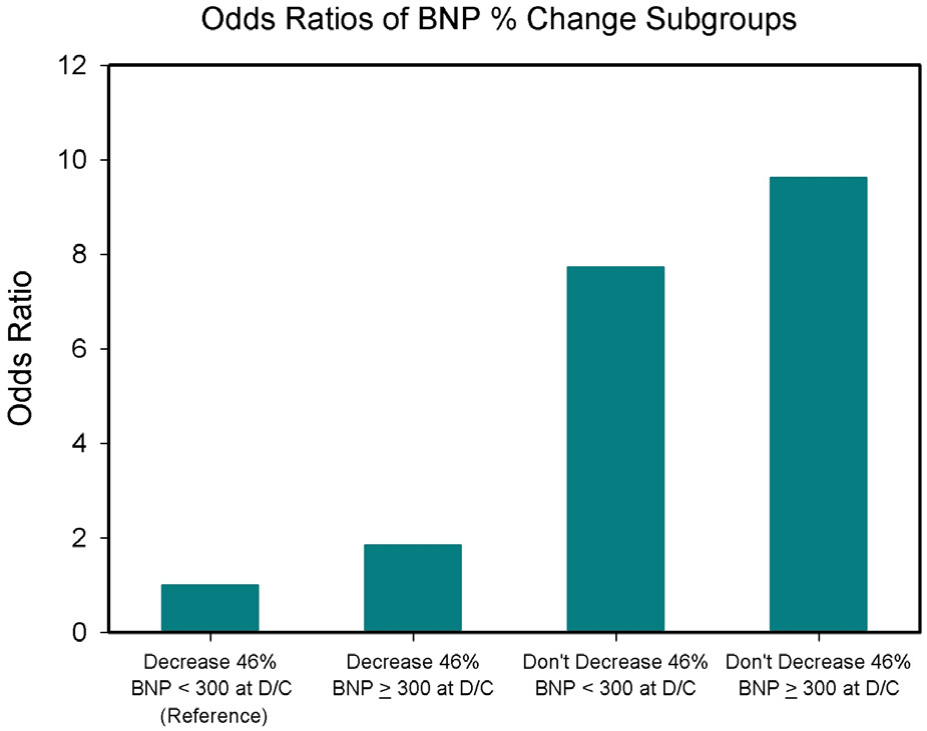
**Odds ratios of BNP precentage change subgroups**. Patients whose brain natriuretic peptide (BNP) values did not decrease 46% and had a discharge BNP value of 300 pg/mL or more had the highest odds ratio for adverse events. D/C: discharge.

Figure 1 shows receiver operating characteristics (ROC) curves for drop percentage of BNP level at 24 hours after hospitalization and for drop percentage of BNP level at discharge. Their AUC are respectively 0.646 and 0.704 (p < 0.001 in both cases). The odds ratio for those with a BNP decrease of <46% (statistically determined by ROC curve) at discharge compared to those with a decrease of at least 46% was 6.18 (95% confidence interval (CI) 3.49 to 10.97, *P *< 0.001). In multivariate analysis including both predictors and their interaction, the interaction term was not significant (*P *= 0.874). For an analysis including both predictors but no interaction term, the OR for discharge BNP 300 pg/ml or above was 1.93 (95% CI 1.03 to 3.59, *P *= 0.039) and the OR for percentage decline in BNP below 46% was 5.06 (95% CI 2.78 to 9.22, *P *< 0.001), indicating that there were additive effects of both predictors. Taking both predictors together a four-group model was developed. The OR of the patients whose BNP level at discharge was above 300 pg/mL and whose percentage decrease at discharge was above 46% compared with those whose BNP level at discharge was below 300 pg/mL and whose percentage decrease at discharge was above 46% was 1.83 (Figures 2 and 3). The OR of the patients whose BNP level at discharge was below 300 pg/mL and whose percentage decrease at discharge was less than 46% compared with the group whose BNP level at discharge was below 300 pg/mL and whose percentage decrease at discharge was >46% was 4.75 (p < 0.002) (Figure 2 and 3). The odds ratio of the patients whose BNP level at discharge was >300 pg/mL and whose percentage decrease at discharge was below 46% compared with the group whose BNP level at discharge was below 300 pg/mL and whose percentage decrease at discharge was above 46% was 9.61 (*P *< 0.001; Figures 2 and 3).

## Discussion

In our previous studies we demonstrated that, in ADHF patients, the clinical improvement evaluated by clinical criteria as reduction in respiratory rate, decrease of limb edema, and pulmonary rales, is coupled with a progressive reduction of BNP levels obtained at hospital discharge [16-18].

This study also confirms our previous results. In fact, in our studied population there was a significant mean decrease of BNP levels at discharge time compared with ED admission.

Making the decision to discharge a patient admitted to hospital for ADHF represents one of the major problems for ED physicians. The decision to discharge a patient is generally based on the clinician's subjective perception of the patient's condition, and thus, readmission rates to the hospital (44% at 180 days) and their associated costs are extremely high [32]. The lack of objective parameters to evaluate achieved clinical stability may lead to two consequences: patients who require more intensive treatment and in-hospital monitoring may be inadvertently discharged or patients may be discharged on inadequate therapeutic regimens. On the contrary, those who could be quickly and safely discharged undergo an unjustifiable long stay in the ED. Clinical congestion is often difficult to assess [33]. A patient's weight changes do not always help and a chest x-ray alone cannot lead a physician to safely discharge a patient [34]. Therefore, we need other complementary tools. Absolute BNP levels can be considered as a surrogate for wedge pressure. It has been shown that decreasing BNP levels are correlated with a decrease in wedge pressure [35]. BNP is rapidly cleared due to the shorter half-life (20 minutes) than the inactive form of NT-proBNP. BNP levels have a 'wet' and 'dry' component. The dry component is related to a euvolemic condition, whereas the wet BNP level correlates with the acute congestion of the patient [32]. Reaching a low BNP value at discharge, bringing the patient as close as possible to his dry BNP level, can reduce the rate of both events and re-hospitalizations. At least three consecutive measurements of BNP values (admission, discharge, and a few weeks later) can help to identify HF patients who have a poor short-term prognosis, as shown recently by Faggiano and colleagues [36]. From our results it seems that at the moment of discharge if the clinical improvement of the patients obtained through adequate treatment during hospitalization time is coupled with a BNP reduction of more than 46% compared with admission value, and the absolute value of BNP is below 300 pg/ml, patients can have lower possibilities to have adverse effects in terms of re-hospitalizations and/or cardiovascular deaths. In our opinion this seems to be a very innovative result, in fact, from the literature no data are available on the percentage reduction of BNP obtained during hospitalization as a predictive value for future cardiovascular events. The clinically significant concentration of BNP for prediction of outcome is uncertain but we decided, based on previously published articles, to divide our patients in two groups depending on whether the BNP was greater or less than 300 pg/ml. So far, only the absolute value of BNP at discharge time has been evaluated for a predictive value [29-31], and from our study the value of 300 pg/ml also justifies the results that patients with an absolute level of BNP below 300 pg/ml will have fewer outcomes. From our data it seems that in patients referred to the ED for ADHF after 24 hours of medications, an early drop in BNP level (≥ 25%) was associated with clinical improvement and an absolute value of BNP below 300 pg/mL can allow the patient to be safely discharged from the hospital. If a patient does not look compensated but he has decreased his BNP value by more than 25% at 24 hours, he should undergo one more day of aggressive treatment and after that, if he reaches clinical stabilization he can, in all probability, be discharged; if not, he should be followed-up very soon by his physician. If after the first day of aggressive treatment, the patient does not decrease his BNP level by more than 25%, it is possible to suggest providing one more day of aggressive treatment. After that, if the patient's BNP absolute value drops below 300 pg/mL and this value seems to mirror a clinical improvement, he can be discharged. If not, other interventions such as home health, close medical observation or devices such as pulmonary arterial catheter, implantable cardioverter defibrillator, and bi-ventricular pacemaker should be taken into consideration. If the patients whose drop in BNP level was less than 25% on the first day of treatment, after an additional day still does not decrease their absolute BNP value to below 300 pg/mL, a pulmonary arterial catheter should be inserted and/or a therapy with inotropes should be taken into consideration.

## Conclusions

In conclusion, we can assess that, for people admitted to hospital for ADHF, serial measurements of BNP levels seem to be useful for a better understanding if the obtained clinical improvement during hospitalization can be clarified by a value of BNP that predicts future cardiovascular events. The optimal times to assess the BNP levels seemed to be at admission, 24 hours after admission and at patient discharge. A greater than 25% reduction of BNP levels 24 hours after admission and a 46% or greater reduction of BNP levels at discharge compared with the admission, together with a BNP absolute value of less than 300 pg/mL, demonstrate a strong negative prognostic value for future cardiovascular outcomes.

Discharge BNP values seem to be a very strong predictor of subsequent outcomes in patients admitted for ADHF and should be used for reducing future cardiovascular events. In any case it must be taken into account that from our study population the clinical criteria of improvement (reduction of dyspnoea, respiratory rate below 30 breaths/min, oxygen saturation above 90%, complete clearance of rales at chest examination, significant reduction of lower limb edema) were the only used criteria to discharge a patient. But, from our observational study, it resulted that if we coupled clinical improvement and evaluation, at discharge time, of BNP percentage changes and absolute value we could have additive information on the prognosis of these patients. Limitations of the study include that in this study we did not investigate what should be done for those patients who failed to achieve the reduction in BNP at discharge. In the future, more intention to treat studies should be properly designed to consider new therapeutic strategies for those non-responsive patients to the traditional treatment in terms of BNP percentage and absolute value reduction.

## Key messages

• In patients with ADHF, serial assessment of BNP at admission, 24 hours after admission, and at discharge time are useful to confirm clinical improvement obtained during hospitalization.

• A reduction of 25% or greater of BNP at 24 hours from hospitalization compared with admission levels has a strong negative prognostic value for future cardiovascular events.

• A reduction of 46% or greater of BNP at discharge coupled with BNP absolute value below 300 pg/ml demonstrate a strong negative prognostic value for future cardiovascular events.

## Abbreviations

ACE: angiotensin-converting enzyme; ADHF: acute decompensated heart failure; BNP: brain natriuretic peptide; CI: confidence interval; ED: emergency department; HF: heart failure; IQR: interquartile range; NT-proBNP: n-terminal pro-B-type natriuretic peptide; NYHA: New York Heart Association; OR: odds ratio; ROC: receiver-operating characteristic.

## Competing interests

SDS and AM have received both consult and have received research financial support from Biosite-Inverness, who is the sponsor for the study. The other authors declare that they have no competing interests.

## Authors' contributions

SDS managed day-to-day activities of the study and wrote the majority of the manuscript. LM, VP, RM, EF, AM, PB, AS, GB, PM, MP, KS, and PC assisted with patient recruitment, analysis, and writing/approving the manuscript. AM helped design the study, secure funding for the project, and oversaw the entire project.
